# Effective Treatment of Rheumatoid Arthritis-Associated Interstitial Lung Disease by B-Cell Targeted Therapy with Rituximab

**DOI:** 10.1155/2012/272303

**Published:** 2012-10-22

**Authors:** Wolfgang Hartung, Judith Maier, Michael Pfeifer, Martin Fleck

**Affiliations:** ^1^Department of Rheumatology and Clinical Immunology, Asklepios Clinic, 93077 Bad Abbach, Germany; ^2^Department of Internal Medicine I, University of Regensburg, 93042 Regensburg, Germany; ^3^Department of Pulmonology and Internal Medicine II, University of Regensburg, 93042 Regensburg, Germany

## Abstract

Rheumatoid arthritis- (RA-) associated interstitial lung disease (RA-ILD) is the extra-articular complication with most adverse impact on the quality of life and survival in RA patients. However, treatment options are limited and controlled studies are lacking. Here, we present the case of a 66-year-old patient suffering from severe RA-ILD, which has been successfully treated with Rituximab (RTX). After failure of conventional DMARD therapy, our patient showed sustained improvement of clinical pulmonary parameters as well as joint inflammation following B-cell depletion with RTX. The six-minute-walk test improved from 380 meters to 536 meters and the forced vital capacity from 2.49 liters to 3.49. The disease activity score could be reduced from 7.7 to 2.8. Therefore, RTX might be considered as an alternative treatment for RA-ILD in patients not responding to conventional DMARD therapy.

Rheumatoid arthritis-associated interstitial lung disease (RA-ILD) is RA's extra-articular complication with most adverse impact on quality of life and survival. However, treatment options are limited and controlled studies are lacking. We report a 66-year-old man suffering from severe RA-ILD. Treatment with methotrexate as well as cyclophosophamide failed to improve the respiratory function. Surprisingly, we noticed a fast and sustained improvement with rituximab. Thus we consider rituximab as an alternative treatment strategy for DMARD-resistant RA-ILD.

A now 66-year-old Caucasian was diagnosed with idiopathic pulmonary fibrosis (IPF) due to persistent dry coughing and dyspnea concomitant with impaired functional capacity of the lung and typical radiographic findings two years ago. A few months later, the initial respiratory symptoms were followed by polysynovitis associated with high systemic inflammatory activity. With regard to the clinical symptoms and presence of high titers of rheumatoid factor (Rf) as well as anticyclic citrullinated peptide (CCP) antibodies, the diagnosis of RA was established. Consequently, treatment with NSAIDs, prednisolone and a course of leflunomide for half a year has been initiated in a community hospital without substantial improvement. Therefore, the patient presented to our outpatient clinic with persistent polysynovitis and severe systemic inflammatory response (DAS28: 7.47; CRP: 77 mg/L).

In light of the previous findings in combination with HRCT (Figure 1) we diagnosed RA-ILD. To obtain histological confirmation, transbronchial lung biopsy was performed, which revealed unspecific fibrotic changes. Since the patient rejected open lung biopsy, we could not specify the histopathologic patterns. MTX (15 mg/week) and prednisolone treatment (30 mg per day) were initiated. We choose MTX because at that time that patient mainly suffered from joint symptoms but presented only marginally impaired lung function. However, the patient subsequently developed pulmonary infection with rapid improvement with antibiotic therapy despite lacking device of an infectious agent. Since we could not exclude MTX-mediated worsening of the lung disease, MTX was terminated after only three applications. With steroids as the remaining therapy, disease deteriorated rapidly with polysynovitis and high levels of systemic inflammation (CRP 120 mg/L), and therefore multiple intra-articular steroid injections were performed. At that time, pulmonary involvement had progressed to a partial respiratory insufficiency with the requirement of home oxygen supplementation. Arterial blood gas analysis (BGA) revealed a pO_2_ of 53 mmHg, a pCO_2_ of 35 mmHg, and O_2_-saturation of 88%. Distance in the 6-minute walk test (6MWT) as a specific measurement for the functional pulmonary capacity was significantly reduced to 380 m (normal values for untrained man: 600–700 m). In addition, the forced vital capacity (FVC) of the lung was reduced to 2.49 l. Despite the lack of evidence in RA-ILD, we decided to install cyclophosphamide (CYC) in combination with high dose prednisolone, which has been suggested as a treatment option in patients suffering from idiopathic pulmonary fibrosis as well as patients with scleroderma-associated lung disease.

However, after three courses of CYC the patient presented with persistently high disease activity (DAS28: 7.7; CRP 72 mg/L), and the respiratory situation had only been slightly improved (Table 1). At this point, we combined CYC with rituximab (RTX). Within the next 12 weeks, profound improvement of joint manifestations in combination with stable pulmonary function could be observed (DAS28: 4.5; CRP 39 mg/L), so that we were able to reduce the prednisolone dose to 7.5 mg daily. Due to this improvement, the patient stopped CYC therapy after a total of 6 infusions by himself and also rejected other DMARD therapy. Eight weeks following reduction of prednisolone to 5 mg per day, RA exacerbated again, and the patient was again admitted to our outpatient clinic with polysynovitis (DAS28: 6.3; CRP 65 mg/L). Immunophenotyping revealed complete peripheral B cell reconstitution, and therefore prednisolone was increased up to 20 mg per day, and a combination therapy of MTX (10 mg/week) and RTX (2 × 1000 mg within 2 weeks) was started. Again, successful depletion of peripheral B-cells could be confirmed by FACS-analysis, and RA disease activity declined within the next 16 weeks (DAS28: 2.8; CRP 1.7 mg/L). Most surprisingly, the pulmonary situation improved dramatically as demonstrated at a follow-up visit 12 weeks after the second RTX course by normal values for the BGA with a pO_2_ of 63 mmHg and a pCO_2_ of 36 mmHg. This striking recovery could be confirmed by an almost normal value for the 6MWT of 563 m. In addition, lung function testing showed a stabilization of the FVC at 3.49 L. Furthermore, the patient's quality of life had significantly improved since home oxygen supplementation could be discontinued (Table 1).

Pulmonary involvement in RA is directly responsible for 10–20% of all mortality [1–4]. Despite this adverse impact solid treatment recommendations for RA-ILD are still lacking. In contrast, drug-induced worsening of pulmonary function in RA patients is well known [5, 6]. Combined CYC and steroid therapy does not substantially improve survival in IPF patients [7]. RA-ILD has similar features with regard to histopathology as well as gene expression profile, but the clinical course seems to be milder. However, in our case we could not definitively exclude a response to treatment with CYC alone but the lack of significant improvement of 6MWT under CYC makes this unlikely.

RTX is known to be an effective therapeutic instrument in joint diseases. Here, we present a remarkable response of RA-ILD to RTX. With regard to the histopathologic finding of peribronchial infiltration with B-lymphocytes in RA-ILD, we propose that B cells are critically involved in the pathogenesis of RA-ILD. We therefore regard RTX as an alternative treatment for RA-ILD in patients with primary failure of conventional DMARD therapy. Nevertheless, further studies are warranted to substantiate our observation, and to enlarge our therapeutic armamentarium for this severe disorder.

## Figures and Tables

**Figure 1:**
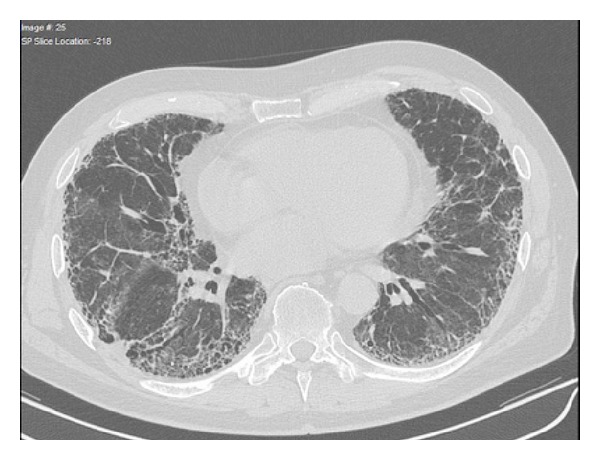
HR-CT-scan of the thorax revealed pronounced signs of pulmonary fibrosis including consolidations and honeycombing especially in basal areas.

**Table 1:** Development of laboratory and clinical parameters in response to different immunosupressive agents.

Parameter	Treatment		
	MTX + prednisolone (baseline)	Cyclophophamide + prednisolone (week 18)	Rituximab + MTX + prednisolone (week 59)
6MWT (% of debit)	380 m (63.60%)	436 m (79.39%)	536 m (90.44%)
FVC (% of debit)	2.49 L (56.8%)	3.36 L (76%)	3.49 L (87%)
BGA			
pO_2_	53 mmHg	62 mmHg	63 mmHg
pCO_2_	35 mmHg	38 mmHg	36 mmHg
DAS 28	7.47	7.7	2.8
CRP	131 mg/L	72 mg/L	1.7 mg/L
Ultrasound synovitis Score (PIP II–V)	10	7	4
